# Research progress and the prospect of using single-cell sequencing technology to explore the characteristics of the tumor microenvironment

**DOI:** 10.1016/j.gendis.2024.101239

**Published:** 2024-02-03

**Authors:** Wenyige Zhang, Xue Zhang, Feifei Teng, Qijun Yang, Jiayi Wang, Bing Sun, Jie Liu, Jingyan Zhang, Xiaomeng Sun, Hanqing Zhao, Yuxuan Xie, Kaili Liao, Xiaozhong Wang

**Affiliations:** a Department of Clinical Laboratory, The 2nd Affiliated Hospital, Jiangxi Medical College, Nanchang University, Nanchang, Jiangxi 330006, China; b Queen Mary College, Jiangxi Medical College, Nanchang University, Nanchang, Jiangxi 330006, China; c School of Public Health, Jiangxi Medical College, Nanchang University, Nanchang, Jiangxi 330006, China; d The Second Clinical Medical School, Jiangxi Medical College, Nanchang University, Nanchang, Jiangxi 330006, China

**Keywords:** Application, Prospect, Research progress, Single-cell sequencing, Tumor microenvironment

## Abstract

In precision cancer therapy, addressing intra-tumor heterogeneity poses a significant obstacle. Due to the heterogeneity of each cell subtype and between cells within the tumor, the sensitivity and resistance of different patients to targeted drugs, chemotherapy, *etc.*, are inconsistent. Concerning a specific tumor type, many feasible treatments or combinations can be used by specifically targeting the tumor microenvironment. To solve this problem, it is necessary to further study the tumor microenvironment. Single-cell sequencing techniques can dissect distinct tumor cell populations by isolating cells and using statistical computational methods. This technology may assist in the selection of targeted combination therapy, and the obtained cell subset information is crucial for the rational application of targeted therapy. In this review, we summarized the research and application advances of single-cell sequencing technology in the tumor microenvironment, including the most commonly used single-cell genomic and transcriptomic sequencing, and their future development direction was proposed. The application of single-cell sequencing technology has been expanded to include epigenomics, proteomics, metabolomics, and microbiome analysis. The integration of these different omics approaches has significantly advanced the development of single-cell multiomics sequencing technology. This innovative approach holds immense potential for various fields, such as biological research and medical investigations. Finally, we discussed the advantages and disadvantages of using single-cell sequencing to explore the tumor microenvironment.

## Introduction

**Figure 1:**
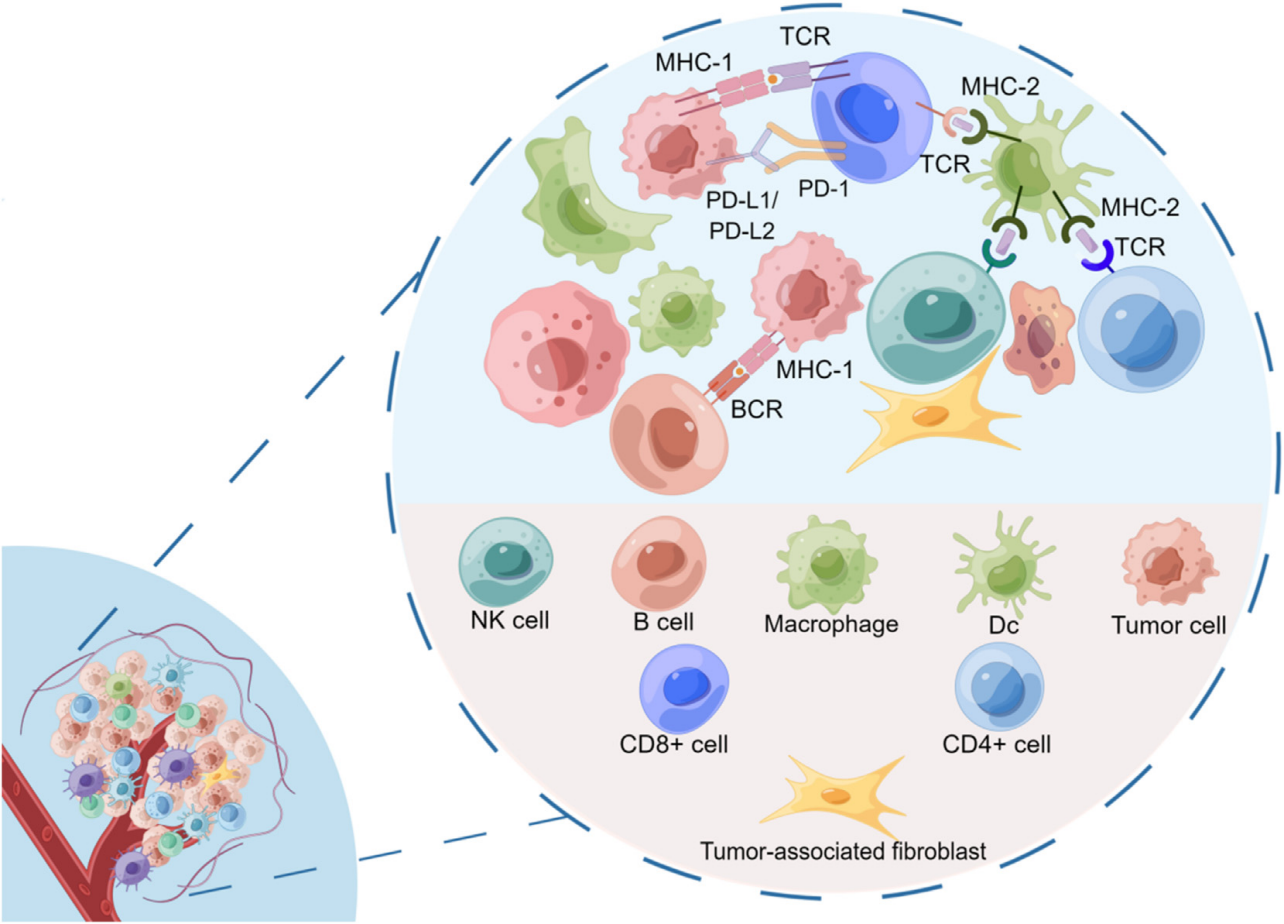
Presentation of cells and related factors in the tumor microenvironment. The figure is made using Figdraw.

The tumor microenvironment comprises tumor cells and the places they depend on, including tumor-associated fibroblasts, dendritic cells, myeloid-derived suppressor cells, cytokines, tumor-associated macrophages, and angiogenic factors (Fig. 1).^1^ Tumor cells and the surrounding microenvironment have a symbiotic relationship, with each component supporting and strengthening the other.^2^ The microenvironment theory proposes that alterations in the local microenvironment of individuals with tumors, such as interstitial remodeling, angiogenesis, an inflammatory environment, reactive oxygen species, and an increase in carcinogenic factors, result in cancer cells being better able to colonize metastatic organs for early-stage preparation and eventually form metastases.^3^, ^4^, ^5^ The tumor microenvironment has physiological characteristics such as hypoxia, low pH, interstitial high pressure, and high vascular permeability, in which there are a variety of stromal cells, regulatory factors, proteases, and other substances, which are important for providing the necessary material basis for tumor occurrence, development, invasion, metastasis, drug resistance, and immune response.

Therefore, in addition to traditional treatment methods, studying the tumor microenvironment is a novel exploration method for addressing tumors. The level of extracellular matrix was shown to be inversely related to the clinical chemotherapeutic response rate, indicating that the tumor microenvironment, as the “soil” of tumors, has an important relationship with tumor progression and metastasis.^6^^,^^7^ Treatments targeting the tumor microenvironment are gradually becoming a research hotspot, including anti-vascular therapy targeting new blood vessels, tumor immunotherapy, and tumor sensitization therapy to reverse drug resistance.^7^^,^^8^ The recipient of the Nobel Prize in Physiology or Medicine was Ralph M. Steinman in 2011 for the “discovery of dendritic cells and their role in acquired immunity”, making it a breakthrough in the treatment of malignant tumors.^9^ Tumors are composed of numerous cell populations. In addition to malignant tumor-transformed cells, the tumor microenvironment may be composed of different infiltrating immune populations (myeloid cells, dendritic cells, lymphocytes, *etc*.), which have a significant impact on the cellular components responsible for providing blood supply to the tumor and other stromal cell populations. During carcinogenesis, genetically heterogeneous populations of different cancer cells undergo the process of emergence, evolution, and interaction with cells in the tumor microenvironment, resulting in manipulation of host metabolism, evasion from immune surveillance, spread to distant body locations, and ultimately death.^10^^,^^11^ Furthermore, several genomic subclones are included in the malignant populations with an aberrant development process, contributing to the existence of multiple lineages of immature, stem-like subpopulations, and more differentiated cells. Analyzing multiple cells of the identical type in the same state can help obtain the exploratory ability to recognize and characterize these complicated cell populations.

The sequencing method adopted by ordinary next-generation sequencing is to perform blind sequencing of the entire tumor tissue to screen for differential genes. This makes it impossible to study the cellular localization of differentially expressed genes. Tumors are complex cellular biological systems. Tumor cells have great heterogeneity in different patients and cells. Blind sequencing of tissues is obviously unable to explain the signaling pathways, target cells, and other mechanisms of their effects. In addition, ordinary next-generation sequencing can only study changes in gene expression but cannot analyze changes in various cells in tumor tissue. This obviously eliminates the understanding and exploration of different cell groups for complex tissue cell groups with high heterogeneity, such as tumor tissue. The research level of single-cell sequencing (SCS) technology focuses on analyzing individual cells, allowing for the isolation screening of single cells as well as identification sequencing of cell populations. Due to its unique cell screening and identification characteristics, it is widely used in tumor heterogeneity and tumor immune microenvironment research.^12^, ^13^, ^14^ The utilization of SCS for DNA and RNA exhibits an exquisite approach to assessing heterogeneity in tumors and predicting tumor-microenvironment interactions.^14^^,^^15^ Remarkably, the utilization of CRISPR‒Cas9 technology in a KRAS mutant mouse model enables the mapping of a comprehensive malignant cell profile through single-cell analysis. This technique effectively addresses the challenges associated with low mutation sensitivity and the inability to interpret the details of changes in tumor subtypes. Likewise, it can help track lung cancer spread patterns and key genes. These new findings demonstrated that lung cancer in people with KRAS mutations might be better managed clinically and treated with targeted therapies.^16^ Another study combining single-cell RNA sequencing (scRNA-seq) and high-confidence clone tracking showed an elaborate description of leukemia stem cells for the first time, providing new insights into understanding leukemia occurrence and treatment.^17^ SCS technology can comprehensively and objectively analyze the cell diversity in tumors, malignant cell subsets, the immune microenvironment, especially tumor-infiltrating lymphocytes, and other important factors affecting the disease process, which provides a theoretical basis for further exploring the tumor microenvironment and looking for potential drug therapy targets and possible cellular mechanisms of tumor patients. In the last few years, SCS technology has scored tremendous achievements and has been extensively utilized in cancer research, but there remain many difficulties and issues to be explored. In this study, we present a comprehensive review of SCS technology's use and the most recent developments in tumor research, focusing on its research progress in exploring the tumor microenvironment.

## Advances in SCS technology in tumor microenvironment research

Heterogeneity within and among tumors is an important feature of tumors, with different cancer cell clonal components at different spatial sites within the tumor leading to spatial heterogeneity within tumor tissues.^18^ With the proliferation and apoptosis of tumor cells, tumor cell subsets also undergo temporal changes during the incidence and progression of cancer, resulting in temporal heterogeneity.^18^ The tumor microenvironment consists of a variety of cell types, including stromal cells, immune cells, and other non-cancerous cells, in addition to cancer cells. This diverse composition contributes to the heterogeneity observed within tumors.^19^ An in-depth comprehension of the tumor microenvironment's makeup and cell–cell interactions is necessary to truly understand cancer progression and the emergence of medication resistance. SCS enables the most accurate dissection of the complicated constitution of tumors as well as the capture of uncommon cell types. In this review, we concentrate on the research progress of utilizing SCS technology to probe the tumor microenvironment.

## Single-cell genome sequencing

Research on the clonal and subclonal formation of primary tumors is an early utilization of SCS in cancer research. Breast, kidney, bladder, and colon cancers, hematological malignancies, and glioblastoma have all been treated by DNA-based SCS.

Single-cell DNA sequencing is predominantly employed in breast cancer research to discern somatic nucleotide variations, copy number alterations, and structural variations.^20^ Currently, the primary approach for single-cell genome sequencing of breast cancer involves utilizing whole-genome sequencing to sequence copy number alterations, which allows for the identification of tumor heterogeneity as well as the clonal organization and progression of the tumor. The study conducted by Navin et al in 2011 involved low-coverage mononuclear sequencing (∼6%) on 100 single cells of multigenomic triple-negative breast cancer (ER-/PR-/Her2-) and 100 single cells of single-genomic triple-negative breast cancer (ER-/PR-/Her2-) along with their corresponding liver metastases. Three separate subclones were observed, which could be considered a common occurrence in the successive expansion of clones. In contrast, in regard to a single genome, there is monoclonal expansion leading to the development of a primary tumor and subsequent metastasis. Analysis of copy number alterations also showed that the original tumor emergence and metastasis were attributed to clonal expansion. Furthermore, a recent pattern in tumor development, known as discontinuous clonal evolution, contradicted the progressive pattern with few intermediates.^21^ The initial study on breast cancer utilizing single-cell whole-exome sequencing was conducted and published in 2014 by Wang et al. The current work used a novel method to validate putative somatic mutations. The method involved conducting highly focused single-molecule sequencing (exceeding 110,000 ×) on a large quantity of tissue.^22^ In colon cancer research, two primary colon tumor populations known as CD45^−^EpCAM^high^CD44^+^ and CD45^−^EpCAM^high^CD44^−^ populations were isolated by Liu et al by utilizing fluorescence-activated cell sorting, followed by low-coverage single-cell whole-genome sequencing at 0.1 × depth, allowing them to distinguish between individual cancer stem cells (EpCAM^high^CD44^+^) and disseminated tumor cells (EpCAM^high^CD44^−^) based on somatic copy number alterations.^23^ Hematological malignancies encompass acute lymphoblastic leukemia and acute myeloid leukemia as notable instances.^24^, ^25^, ^26^ The genesis and subsequent clonal development of common cancer cells can be inferred from studies that have demonstrated shared mutations between various cancer cell clones in individual cancer patients.

## Single-cell transcriptome sequencing

### Single-cell transcriptome sequencing in immune cells

**Figure 2:**
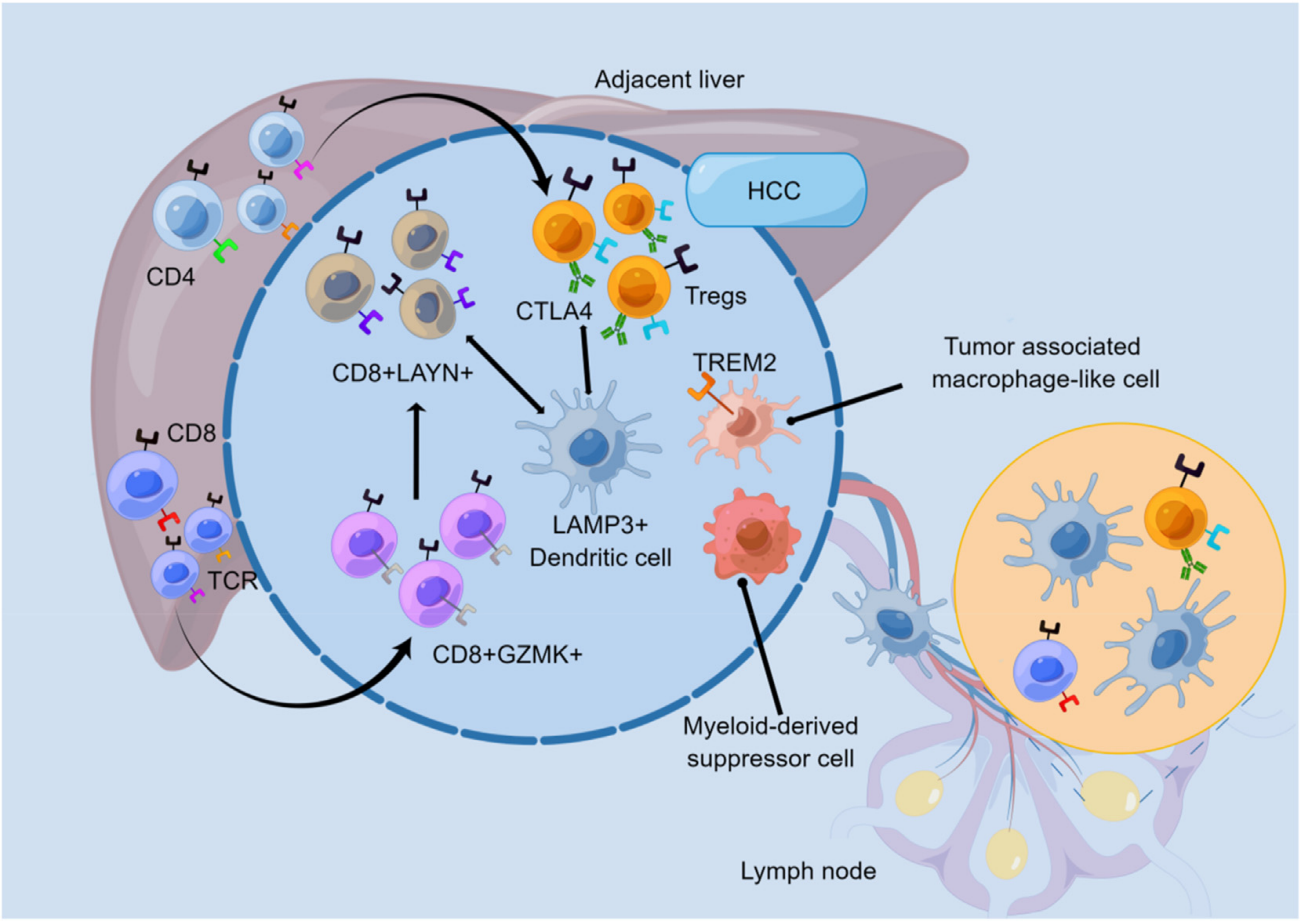
Schematic diagram of the complicated immune cell microenvironment in hepatocellular carcinoma (HCC) by single-cell RNA sequencing analysis. The figure is made using Figdraw. Adapted from figure 7 of https://doi.org/10.1016/j.jhep.2020.06.004.

scRNA-seq holds immense importance in the discovery of previously unknown immune cell types or heterogeneity within the same immune cell population.^27^ The components of the immune cell microenvironment of hepatocellular carcinoma (HCC) were analyzed by scRNA-seq. In 2017, Zheng et al found that immunosuppressive regulatory T cells and CD4^+^CTLA4^+^ originating from CD4 T cells were clonally enriched.^28^ Meanwhile, exhausted CD8^+^LAYN^+^ lymphocytes, different from CD8^+^GZMK^+^ lymphocytes that originated from cytotoxic CD8^+^ T cells, were clonally enriched in HCC patients.^29^ Mature lysosomal-associated membrane protein 3-positive dendritic cells were found in the lymph nodes and HCC tissue, and they interact with relevant T cells and natural killer cells, causing the induction of dysfunction in T-cell activity.^29^ Macrophages in HCC behave in a myeloid-derived suppressor cell-like immune cell function regulator state and tumor-associated macrophage-like state, which expresses TREM2 and GPNMB^30^, ^31^, ^32^ (Fig. 2). In June and October 2018, Professor Zhang Zemin's team published major research results in *Nature Medicine*^33^ and *Nature*,^34^ respectively. T-cell immunoprofiling of lung and colorectal cancer was performed at the individual cellular extent, revealing the categorization of T-cell subsets, tissue distribution features, heterogeneity of the intratumor population, and gene expression that drugs are targeted to in lung cancer and colorectal tumors, and identifying possible state shift links between T-cell subsets and subsets across various tissues, which has important implications for lung cancer and colorectal cancer diagnosis and treatment. On August 23, 2018, researchers at the Memorial Sloan-Kettering Cancer Center in the United States conducted a single-cell transcriptome sequencing analysis on 47,016 immune cells from human breast tumors. The gene expression characteristics of these cells were then compared to those of matching normal breast tissues, peripheral blood, and lymph nodes.^35^ Intra-tumoral lymphocyte and myeloid cell heterogeneity were revealed, showing significant phenotypic expansion compared with healthy breast tissue. This diversity arises from the activation of different combinations of genes due to exposure to diverse environmental cues, with specific involvement of the T-cell receptor in shaping T cells' combinatorial gene expression. The continuum of changes in T-cell status that has been observed subverts the previous classical concept of a tumor microenvironment formed by less differentiated or activated discrete states. In December 2018, a detailed immune cell map of melanoma was created by Dr. Li Hanjie and colleagues from the Ido Amit Laboratory in Israel.^36^ They utilized single-cell transcriptome sequencing and performed single-cell T-cell receptor sequencing analysis on immune cells obtained from 25 patients with melanoma. The study found that although different immune cell subgroups were present in the majority of patients, their relative abundance varied greatly among patients. In addition, the differentiation pathways observed for CD8 T cells are highly conserved despite the differences in abundance.^36^

### Single-cell transcriptome sequencing in tumor cells

Single-cell transcriptome sequencing of tumor cells is used to study tumor evolutionary patterns and supports tumor stem cell models.

The utilization of scRNA-seq in glioma^37^ showed that the heterogeneity of tumors could be attributed to the cellular differentiation process of neural stem cells. Therefore, it can facilitate the construction of a model for cancer stem cells. In particular, research on intra-tumoral variation in colorectal cancer^38^ combined single-cell technology with tumor organoid culture showed a significantly higher frequency of somatic mutations in cancer cells than in normal cells. The study's authors also discovered the appearance of the most mutations in the process of the final dominant clonal expansion, resulting from a mutational process lacking in normal controls.

Roerink et al^38^ studied the characteristics of the tumor structure of clones and subclones to obtain a pattern of cancer evolution, indicating the underlying dynamics of cancer development. These findings demonstrate the specific ability of SCS in researching tumor cell polymorphisms, which leads to different evolutionary patterns among tumors. Significantly, the single-cell data show that sustained proliferation and clonal expansion occur in most tumor cells, thus challenging the tumor stem cell pattern. Furthermore, the observation that cell differentiation promotes tumor heterogeneity was indicated by scRNA-seq data to help support the cancer stem cell model. Copy number alterations and point mutations in tumor cells have disparate developmental patterns; the first tends to be intermittent, and the latter tends to accumulate gradually. Resolving outstanding discrepancies will be necessary before applying matching models of tumor initiation and development to various cancers. The various models of cancer evolution need to be coordinated by researchers with a larger number of specimen sizes and higher molecular and cellular precision. The study sequencing single-cell organoids should have larger specimens and other tumor types to provide templates for studying cancer development.

Within the intricate milieu of tumor tissue, single-cell transcriptome sequencing has the capability to differentiate between malignant and non-malignant cells. Using scRNA-seq, large copy number variations and aneuploidy may be quickly identified and used to discriminate between cancerous and benign tumor cells.^39^ Xiao et al^40^ dissected glioblastoma cell composition and employed the previously reported computational pipeline to generate copy number variation-based malignancy scores that were then utilized to identify glioblastoma cells. Malignant cells score higher in malignancy than normal oligodendrocytes with various copy number variations. In addition, marker genes were passed along by non-tumor cells. Compared with non-malignant oligodendrocytes, tumor cells frequently have chromosome 10 deletion, which is one of the earliest and most frequently observed genetic abnormalities in adult glioblastoma. There are genetically heterogeneous malignant cells present in all glioblastoma patients, as evidenced by their own, primarily unique copy number variation subclones.

### Single-cell transcriptome sequencing of the expression of surface markers

An article published in *Nature Immunology* in 2016 found that CD127^+^ innate lymphocytes in the small intestine and tonsils of humans had apparent heterogeneity by analyzing the expression of surface markers.^41^ By sequencing the transcriptome of individual γδT cells obtained from peripheral tissues and tumor organizations in mice, scientists can understand how γδT cells enter tumors and play their roles. Notably, γδT cells are heterogeneous within tumors compared with peripheral normal tissues. In addition, nine days of early observation of mouse tumor tissues showed that ten clusters were divided according to the differences in expression levels between cells, and clusters different from peripheral tissues existed in tumor-infiltrating γδT cells, in which cluster 1 and cluster 5 were specifically increased in tumor tissues. This experiment provided a preliminary introduction to the diversity of γδT cells within the tumor microenvironment,^42^ and there was a correlation between tumor immune evasion and the expression of genes exclusively found in severely compromised immune cells. Recent single-cell findings from 10 × genomics in lung cancer showed^43^ that six clusters could be further divided into tumor-rich B cells, with elevated levels of CD20, CXCR4, and HLA-DR being characteristic of two follicular B-cell clusters. In comparison, two populations of plasma B cells exhibited immunoglobulin gamma expression, and the presence of immunoglobulin A, M, and JCHAIN molecules characterized the remaining two populations of B cells derived from mucosa-associated lymphoid tissue. Mass spectrometry also described macrophage subtypes.^32^ In particular, single-cell studies aimed at various cancer types have concentrated on the capacity of T lymphocytes to detect particular cancer neoantigens and destroy tumor cells.^35^^,^^44^^,^^45^ Various T-cell subgroups reside within the tissues of hepatocellular, pulmonary, and mammary cancers, and there is a relationship between lower levels of T-cell depletion and better prognosis.^35^^,^^45^ These immunotherapies result in a variety of cancers by blocking immune checkpoints and adoptively transferring neoantigen-specific T cells to restore cytotoxic T cells. The presence of particular T-cell clusters exhibiting inhibitory capabilities was observed in untreated cancers and those that respond to immunotherapy.^28^^,^^46^^,^^47^ The prediction of patient response to cancer immunotherapy can be made by attractive biomarkers provided by these T-cell cluster marked genes, such as Layilin, which is found in depleted CD8^+^ T cells and hepatoma regulatory T cells, and these biomarkers may become novel targets for further research. However, along with these enormous accomplishments and achievements, a limitation in single-cell studies of the cancer microenvironment is characterizing only the spatial, temporal, and interactional features between tumor and immune cells.

Cancer-associated fibroblasts (CAFs), in addition to the immune cells themselves, are essential for cancer immune evasion and metastasis. Several studies have displayed the heterogeneity of CAFs in multifarious cancer types.^28^^,^^47^ Particular collagen and other extracellular matrix elements were expressed by five different kinds of tumor-resident fibroblasts in 10 × genomics lung cancer research. Two distinct CAF subtypes were found in colorectal cancer detected by SMART-seq2^48^; one contained genes associated with the epithelial-mesenchymal transition, aligning with previous studies on pulmonary cancer. The identification of heterogeneity in these CAF subsets suggested a potential functional role, which aligned with previous findings in metastatic melanoma and head and neck cancer. Interestingly, a specific subset of CAFs specifically expressing numerous complement factors, including C1S, C1R, C3, C4A, CFB, and C1NH (SERPING1), has been observed to be associated with infiltration of T cells, according to an inference of information from The Cancer Genome Atlas project. The investigation of potential mechanisms underlying the impact of CAFs on T-cell recruitment is warranted, although correlation cannot imply causality. In addition, there was a discovery that certain CAFs observed in single-cell studies focusing on cancer affecting the head and neck region were colocalized with cancer cells that have high expression of the p-EMT (partial epithelial-mesenchymal transition) gene program related to metastasis. CAFs have many ligand‒receptor mutual effects on the corresponding tumor cells to support colocalization, which expands new ways to research the mechanisms of tumor invasion. Therefore, we should further study the constantly changing characteristics of CAF gene expression.

## The application prospects of SCS in cancer research

### Clinical application prospects

Single-cell technology uses a tiny amount of tissue to explore tumor heterogeneity and therefore has a high possibility in cancer clinics for the purpose of diagnosing, predicting outcomes, detecting early signs, evaluating risks, monitoring progression, and forecasting treatment response. In the early stages of cancer development, doctors can separate individual cancer cells from blood samples, allowing early detection and assessment of cancer. It can be inferred that cancer cells have a clonal expansion by observing a cluster of known driver mutations solely in many individual tumor cells and then combined with other diagnostic tests to verify the inference, which may require further monitoring or treatment. For a patient who receives a diagnosis of cancer, clonal and subclonal information about the genomic and transcriptomic signatures of the tumor lesion can be revealed by SCS, allowing clinicians to determine the most appropriate treatment. Circulating tumor cells (CTCs) or disseminated tumor cells are sampled longitudinally, and the response to prescribed therapy can also be monitored by SCS.^49^ CTCs are highly viable and have high metastatic potential when entering the blood circulation system. Generally, high-throughput sequencing analysis shows overall genomic characteristics but ignores tumor heterogeneity. Therefore, there arises a necessity for SCS analysis. The utilization of SCS technology can solve the crucial problems of cancer research and has a prominent solution to the origin and development of cancer. In nature biotechnology, smart-seq, a single-cell whole transcriptome amplification method, has been applied in the research of melanoma CTCs.^50^ In 2014, scRNA-seq of breast cancer cases found that a single CTC and mucoglobin formed a CTC cell cluster. They are beneficial to the metastasis of tumor cells.^50^

For example, scRNA-seq technology can improve our understanding of the metastatic characteristics and dynamic changes of CTCs in hepatocellular carcinoma. CTCs can demonstrate hematogenous dissemination and metastasis of HCC. An RNA sequencing library was constructed from 113 CTCs from 10 HCC cases. The expression profiles of HCC primary cancer and CTCs were compared, the differential genes of CTCs relative to primary cancer were conveniently detected, the CTC subpopulation was identified, the characteristic genes and structural characteristics were compared, and potential high-risk tumor metastasis factors were discovered. It was found that CTCs have a higher ability to migrate and invade and that CTCs contain two cell subsets, one of which is a number of genes associated with tumor development, such as platelet-related genes and tumor escape genes, and the other of which has a low degree of malignancy and is in the cell division stage.^51^

Detecting the effectiveness of drugs on multifarious cancer unlimited reproduction suggests that the occurrence or extension of drug-resistant cancer unlimited reproduction can be revealed by the resulting genomic and transcriptome information.^52^ Relevant information for prognostic analysis can be obtained through SCS data, which includes metrics related to rare genomic mutations or gene expression patterns. For instance, the design of multifarious indicators of tumor heterogeneity can be used to forecast response to treatment, probability of the spread of cancer to other parts of the body, period without illness, and overall life expectancy.^53^, ^54^, ^55^, ^56^

### Application prospects of emerging SCS technology

With the continuous development of technology, significant advancements have been achieved in SCS technology in the fields of epigenomics, proteomics, metabolomics, and microbiome, and their combined application with single-cell genomics or transcriptomics has developed single-cell multiomics technology. This allows for more comprehensive cell information, revealing the interrelationships between multiple omics features of cells and further in-depth study of cell function and regulatory mechanisms. This technique has broad application prospects in biological research, medical research, and other fields.

### Single-cell epigenomics

Single-cell epigenomics technologies have matured and steadily entered cancer research.^57^^,^^58^ Single-cell epigenomics describes the molecular layer linking the genome and its functional output. Single-cell epigenomics technology is used in cancer studies, which will help scientists deeply comprehend the regulatory mechanism of the cancer cell phenotype and supply new anti-tumor treatment targets. SCS may also lead to a further understanding of mutagenesis mechanisms in cancer cells, as epigenomics is essential for the stability and dynamics of chromosomes. Halbritter et al^59^ investigated epigenomic diversity in primary Langerhans cell histiocytosis (LCH) lesions, revealing epigenomic heterogeneity among LCH cells. They discovered several recurring LCH cell types through the utilization of scRNA-seq and immunohistochemistry. Furthermore, the presence of actively dividing LCH cells was confirmed, and the distinct subsets were characterized by examining their epigenomic and gene regulatory foundations using chromatin accessibility profiling. This indicates that the heterogeneity of cancer cells is expected to be resolved through single-cell epigenetics studies, providing new ideas for the internal regulatory mechanisms within tumor cells and potential pharmaceutical targets, with the aim of effectively regulating the development of tumors. In addition, the utilization of this technology also promotes the investigation of the regulatory mechanism of tumor-infiltrating cells, thus contributing to the development of targeted tumor microenvironment therapy.

### Single-cell proteomics

Proteins are the main substances that perform cellular functions, so decoding expressed proteins at the single-cell level facilitates the exploration of functional differences in cells. Although single-cell proteomics is still a young science, it is developing rapidly. The technique of mass cytometry by time of flight utilizes the interaction between metal isotope-labeled antibodies and particular signal molecules on or within cells for immunolabeling and enables the study of 100 proteins at the single-cell level.^60^ The application of imaging plasmid cytometry allowed simultaneous analysis of up to 40 protein markers, providing insight into their spatial architecture and interactions, which is based on immunohistochemistry of metal-labeled antibodies and mass cytometry by time of flight. It is easily used clinically because paraffin-embedded tissue samples can be used to perform the procedure.^61^ Using liquid chromatography‒mass spectrometry, we can analyze proteins quantitatively. To improve data stability and detection effect, Wang et al developed a liquid chromatography‒mass spectrometry detection technique to perform targeted quantitative analysis, focusing on the measurement of 17 specific lipids.^62^ Recently, Su et al developed FUNpro, a functional single-cell proteomic profiling tool based on microscopy. Even when phenotypes of cells are dynamic or uncommon, FUNpro is capable of real-time screening, identification, and isolation of single cells of interest.^63^ With the continuous progress and improvement of technology, single-cell proteomics will gradually become as widely used and accurate as other technologies, such as single-cell transcriptome sequencing.

### Single-cell metabolomics

The metabolome refers to a comprehensive collection of small molecule metabolites, such as proteins, nucleic acids, lipids, and others, in a particular cell, organ, or organism. The field of metabolomics can be categorized into targeted or non-targeted metabolomics.^64^, ^65^, ^66^ The combination of the two assays is a potent technique for studying disease-related biomarkers due to the dependability of targeted metabolomics and the high metabolite coverage of non-targeted metabolomics.^67^, ^68^, ^69^

Wang et al^62^ used a minimally invasive method combining machine learning and lipidomics to explore selected lipid changes in tissues of patients suffering from pancreatic ductal adenocarcinoma, and it is expected to serve as an early detection method for pancreatic ductal adenocarcinoma. Improving lung cancer detection is critical to promote the survival rate of lung cancer patients. Subsequently, Wang et al performed SCS of distinct early-stage lung cancers, conducted lipidomic profiling of lung plasma, and discovered that nine lipids in individuals who suffer from early lung cancer could be considered important features for early lung cancer detection.^70^ Single-cell metabolomics is a burgeoning and swiftly evolving area that complements the advances in single-cell analysis in genomics and proteomics. Given the abundant sources and pathways of metabolites, the integration of this metabolic information with SCS technology is expected to reveal more pathological metabolic processes.

### Single-cell microbiomics

The microbiota within tumors plays a crucial role in shaping the tumor microenvironment across different forms of human cancer.^71^ Bulk tissue analysis is commonly used to study the host microbiota in tumors. However, it is limited by the lack of spatial distribution and the local role of the microbiota in tumors.^72^ To solve this problem, Bullman et al invented a scRNA-seq technology called INVADEseq to detect bacteria associated with host tumor cells and somatic cells and unmask alterations in transcriptional pathways associated with tumor progression. They found that the arrangement of the microbiota within a tumor exhibited a well-structured distribution in microniches that serve immune and epithelial cell functions, thereby expediting the development of cancer.^73^ The development of this method reveals the non-negligible impact of the microbiome on tumorigenesis and provides new ideas for better targeted tumor therapy.

### Single-cell multiomics

Single-cell multiomics sequencing enables the simultaneous analysis of several omics data, including an individual cell's genome, transcriptome, epigenome, and proteome.^74^ The development of this technology provides us with a more comprehensive tool to gain insight into cellular functions and regulatory mechanisms. The application prospects of single-cell multiomics sequencing technology in cancer research are substantial. First, reconstructing genetic lineages and tracking their epigenome and transcriptome dynamics can be achieved by scTrio-seq technology developed by Bian et al.^75^ It enables simultaneous assessment of genomic, transcriptomic, and epigenetic data of a single cell, revealing a consistent genome-wide DNA methylation level within a single genetic sublineage, and the genome-wide DNA demethylation pattern is consistent in cancer cells. Second, CITE-seq, which combines scRNA-seq with protein sequencing to concurrently gather gene expression and protein expression information of single cells, can help us obtain greater knowledge about the heterogeneity within the tumor immune microenvironment. Wu et al used this technology to sequence the transcriptome information and epitopes to obtain the immune typing of each immune cell in the tumor microenvironment of breast cancer and finally drew a high-resolution immune map, which provided a basis for the immunotherapy of breast cancer.^76^ In addition, the integration of scRNA-seq and metabolite sequencing enables the simultaneous acquisition of both gene expression and metabolite level information of a single cell, which helps comprehend the cellular biological process and metabolic regulatory mechanisms, discovering new metabolic pathways and metabolic regulatory networks, and studying the adaptability and response mechanism of cells.

In summary, single-cell multiomics sequencing technology possesses broad application prospects in cancer research. It can aid in the research of the tumor microenvironment, the functionality and condition of tumor cells, and the heterogeneity of tumors. The significance of single-cell multiomics sequencing technology in tumor research will continue to increase due to further technological advancement and maturation, offering more precise tactics and techniques for the prevention, detection, and treatment of malignancies.

### Application prospects of single-cell spatial transcriptome sequencing

Although it has a bright future, SCS has significant technical difficulties that restrict it from fully exerting function in studying cancer and clinical applications. The loss of spatial information from testing individual cells during isolation will be a challenge for scientists to address, as the spatial localization of multiple cancer cells and their interactions with tumor microenvironment cells may be crucial in understanding tumor progression, metastasis, the evasion of the immune response, and the development of treatment resistance. Currently, combining imaging techniques with scRNA-seq has resulted in significant advances in this research field. The application of single-cell spatial transcriptome sequencing technology in cancer research involves integrating the spatial information of cells with scRNA-seq technology to infer the spatial structure of tumors. Although it is not yet broadly available, it is useful for cancer biology and therapy. Fluorescence *in situ* hybridization, single-molecule fluorescent *in situ* hybridization, immunohistochemistry, laser capture microdissection, laser scanning microscopy, or *in situ* sequencing can all be used to record the spatial details of individual cells or significant “anchor genes" and subsequently measure or calculate the spatial structure of individual cells to uncover the spatial heterogeneity within the tumor microenvironment. Advanced NICHE-seq technology^77^ supplies a formidable way to study tumor immunology in animal models by allowing separate immune cells for SCS in specifically designated niches of model animals. It will take time for NICHE-seq to be widely used in clinical samples because two-photon laser scanning microscopy needs optical labeling of target cells, which is currently feasible only on model animals.

The clinical application of this technology provides strong support for the clinical targeted therapy of cancer. For example, multiplexed ion beam imaging and spatial SCS of a variety of human skin cancers (cutaneous squamous cell carcinoma) and corresponding normal skin were performed to determine the cellular constitution and structure of cutaneous squamous cell carcinoma, revealing that tumor-specific keratinocytes are the hub of cell-to-cell exchange. Scientists have observed many potential immunosuppressive characteristics, including the colocation of regulatory T cells and CD8 T cells in the stroma of compartmental tumors. Eventually, single-cell characteristics of human tumor xenografts and *in vivo* CRISPR screening were used to confirm the significance of specific gene networks enriched in tumor subpopulations during carcinogenesis. These details pinpoint the stromal cell and cutaneous squamous cell carcinoma subgroups, the geographical niches they interact with, and the communication gene networks related to tumors.^78^ The team from Aoki, Japan, conducted scRNA-seq on 127,000 multicellular cells derived from 22 Hodgkin lymphoma tissue samples and 5 corresponding lymph nodes, providing the first analysis of the phenotypic characteristics of the immune microenvironment specific to Hodgkin lymphoma at the single-cell level. A new Hodgkin lymphoma-associated T-cell subset with prominent suppressant receptor LAG3 expression was recognized by single-cell expression profiling, and this LAG3^+^ T-cell population-mediated immunosuppression was ascertained by functional analysis. An increase in LAG3^+^ T cells directly proximal to MHC class II-deficient carcinoma cells is demonstrated by mixed spatial evaluation of immunocytes in the microenvironment.^79^ These techniques offer a variety of ways to develop DNA methylation conditions, chromosomal availability, protein combining, and higher-order chromosome conformation.

Spatial SCS can be combined with a microarray-based spatial transcriptomics method, and then a multimodal intersection analysis method can be introduced. One study of primary pancreatic tumors performed multimodal crossover analysis, revealing spatially constrained enrichment of ductal cells, macrophages, dendritic cells, and tumor cell subsets as well as apparent co-enrichment with other kinds of cells.^80^ In general, innovative technologies in basic research have progressed rapidly in recent years. Despite their apparent advantages and disadvantages, they provide groundbreaking tools to probe the tumor microenvironment at single-cell resolution. Moreover, experimental methods are often ahead of the corresponding expansion of novel computational and analytical tools. Novel SCS data sometimes break the analytical concepts of batch sequencing investigations, together with novel spaces or features, making actual analytical instruments waste or insufficient. Computer techniques with greater processing capacity are required to effectively analyze increasing amounts of SCS data while maintaining equivalent analytical properties. Spatial scRNA-seq technology has yielded unparalleled data types as well, with two new algorithms newly presented,^81^^,^^82^ allowing the analysis of spatial variation in cancer. The development of computing, particularly for single-cell data, will become an area of interest in the coming years, as there are many challenging but essential issues.

## The advantages and disadvantages of SCS technology in exploring the tumor microenvironment

**Figure 3:**
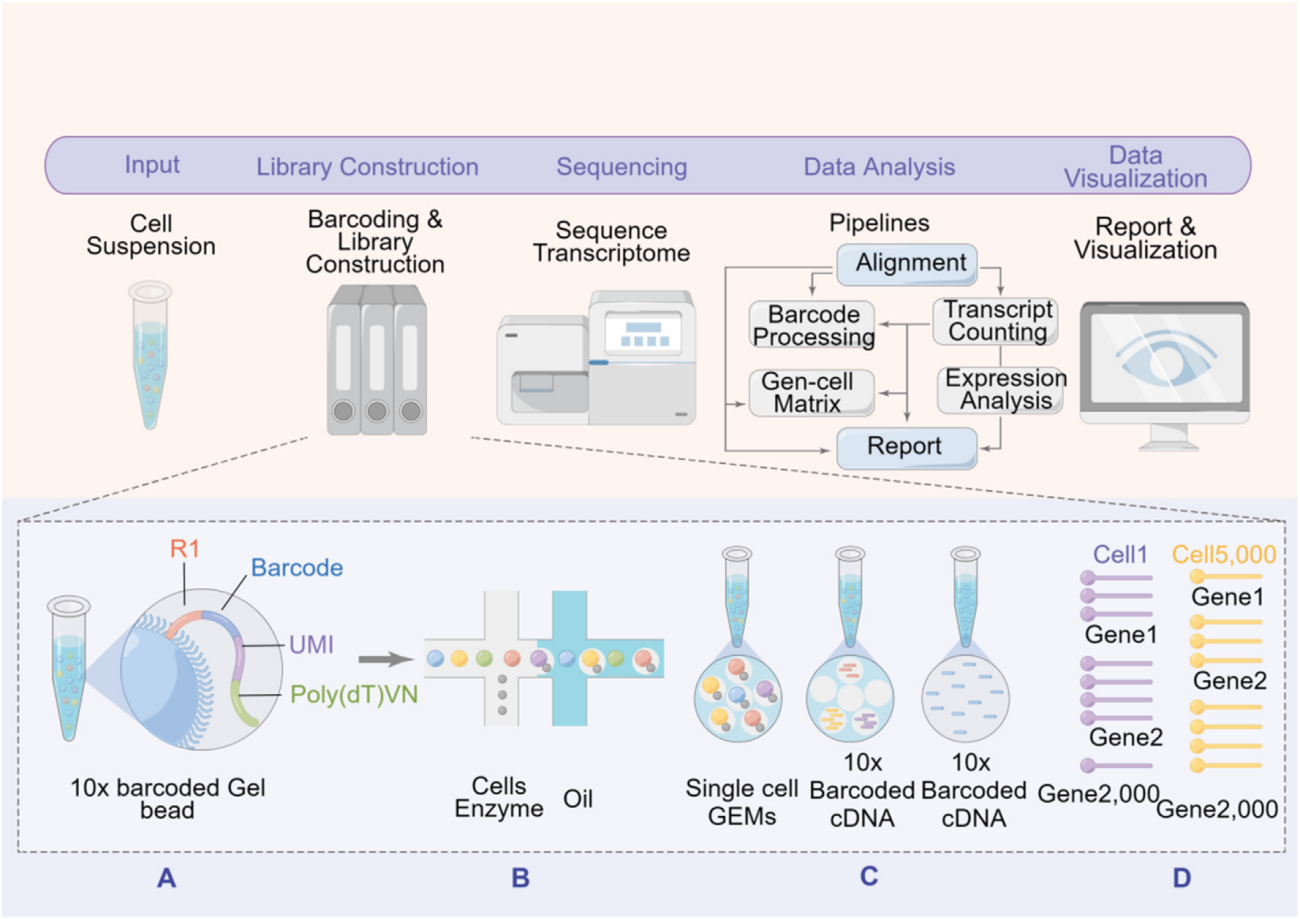
Schematic diagram of 10× genomics chromium system. The figure is made using Figdraw.

SCS technology has made remarkable achievements in the last few years. Platforms such as BD, 10 × , and microwells ensure the isolation of single cells (Fig. 3). Various methods can be employed to acquire and elongate individual cell nucleic acids to facilitate subsequent high-throughput sequencing by making adjustments to the basic techniques of single-cell library construction in molecular biology and chemistry.^83^, ^84^, ^85^, ^86^, ^87^, ^88^, ^89^, ^90^, ^91^ The utilization of SCS technology is widely employed by the immune system, helping us analyze immune cells and explore the tumor microenvironment more comprehensively.^85^ Guo Guoji's team used a set of microwell SCS detection technologies developed by the laboratory to perform single-cell transcriptome sequencing of over 400,000 cells in nearly 50 kinds of tissues and organs in mice and drew the world's first mammalian cell map. This technology enhances the detection efficiency of single-cell technology while significantly reducing costs compared with oil droplet-encapsulated SCS technology. In addition, the throughput of SCS technology has achieved great progress. Several methods allow synchronous sequencing of numerous thousands of single cells,^92^, ^93^, ^94^, ^95^ and methods that combine other experimental methods with SCS technology have been gradually applied^96^ to obtain a more comprehensive analysis of single cells. Topographic SCS was invented to explore spatial information about cell location, which can spatially measure the particular characteristics of various tumor cells.^94^ Different from traditional sequencing technologies, SCS technology takes the unique features of individual cells and the heterogeneity between cells into account, and it helps us understand cell types and functions more accurately. However, SCS also has limitations. Problems such as inaccuracy of results, poor repeatability, weak stability, and lack of evaluation and verification technology have emerged. Amplified by the BD Rhapsody platform, the cDNA activity is high, and the yield is large.^97^ High-throughput nucleic acids can be obtained from single cells, which is beneficial for biological whole-transcriptome analysis and high-sensitivity RNA analysis.^98^ Through SCS on 10× and BD platforms, low-transcription proteins can be detected, further improving phenotypic specificity and facilitating high-throughput cell protein and RNA analysis, which can enhance the understanding of cell diversity. Additionally, SCS on both platforms helps identify cell types and improves resolution, driving biomedicine forward.^99^ The tumor microenvironment encompasses not only cancer cells but also the entirety of a solid tumor. Along with genetic and non-genetic heterogeneity within tumor clones, the microenvironment's heterogeneity between immune cells and stromal cells that penetrate the tumor plays a vital role in various aspects, such as tumor development, angiogenesis, immune escape, spread to other parts of the body, and response to miscellaneous therapies, and influences the overall reaction. By employing high-throughput DNA sequencing, it becomes challenging to differentiate the genomes of these cells within the microenvironment from those of healthy tissues. This poses a significant obstacle in accurately detecting tumor copy number aberrations and point mutations, as it directly impacts the determination of tumor purity. By batch RNA sequencing, there is a mixture between the mRNA of these cells and the mRNA of tumor cells, posing difficulty in distinguishing between their expression signals. The identification of tumor-infiltrating cell types can be deduced by computational deconvolution analysis using RNA-seq data obtained from a large number of tumors.^100^, ^101^, ^102^ Nevertheless, the effectiveness of the algorithms is constrained by the validity of gene signatures across different cell types and being at the same position on the gene signature profiles. SCS can surmount these barriers. By employing scRNA-seq, researchers can analyze the tumor microenvironment in various types of cancers, including melanoma, breast, glioblastoma, colorectal, liver, and lung cancer, and map the associated tumor cell atlas with unprecedented resolution.

**Figure 4:**
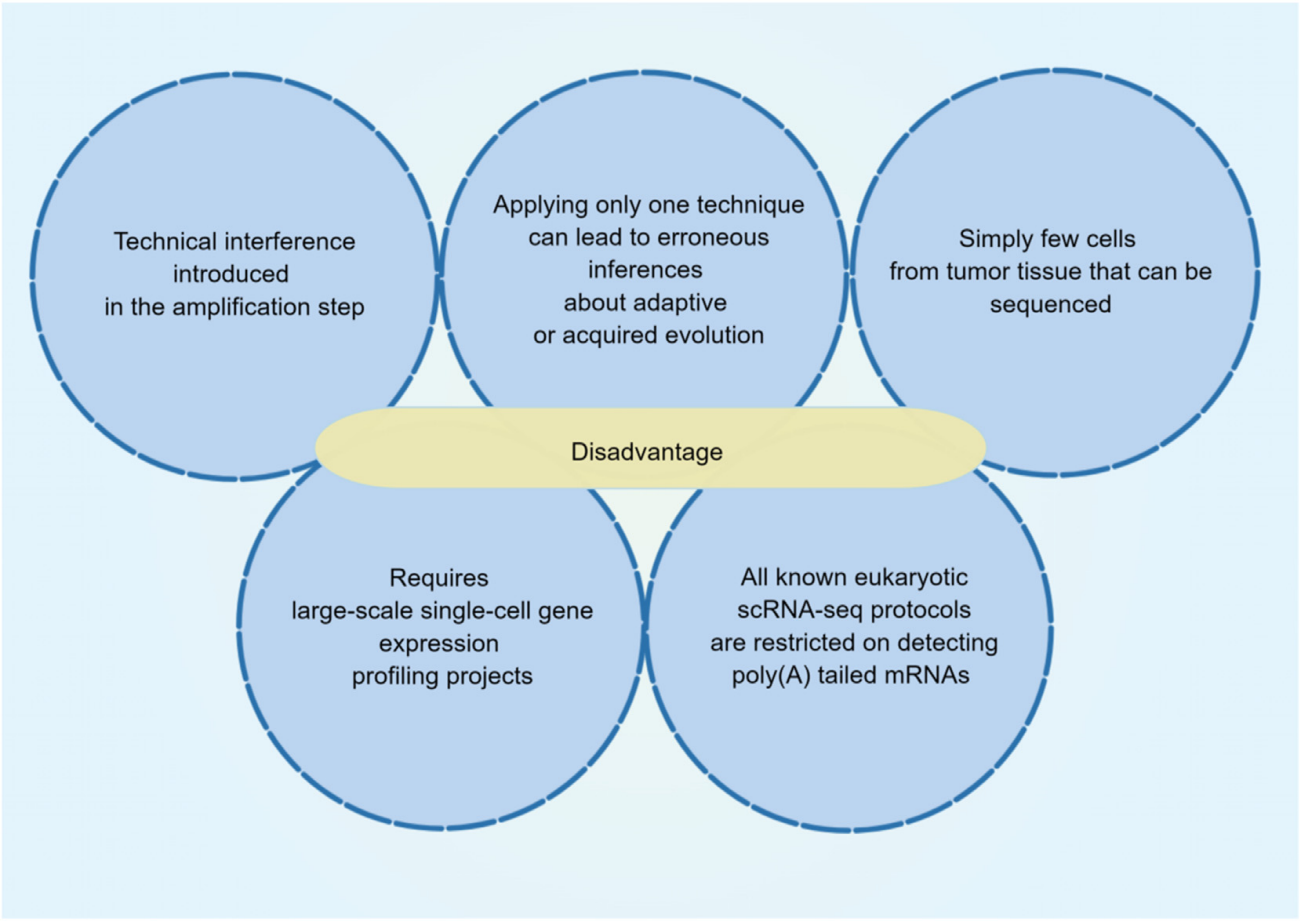
Disadvantages of single-cell sequencing technology to explore the tumor microenvironment. The figure is made using Figdraw.

Despite tremendous progress, SCS still has significant limitations and challenges (Fig. 4). The first challenge is the technical interference introduced in the amplification stage. Accurate identification of single nucleotide variants at the genomic or exomic level is hindered by significant allelic deletions (*i.e.*, selective amplification and sequencing of a single allele from a particular gene in cells with diploid/polyploid genomes) and the uneven coverage of the genetic material. The LIANTI (linear amplification by transposon insertion) approach can partly lower these restrictions.^103^ Likewise, in scRNA-seq analysis, genes with low expression levels, even when detected, can be compromised owing to shedding and are easily affected by technical noise, although they often encode proteins that play a role in regulation and signaling. Although many computational methods are available for simulation or estimation,^104^^,^^105^ their performance varies and may introduce human bias.

Simply a few cells from tumor tissue that can be sequenced is the second challenge. Large chunks of tissue are composed of millions of cells. However, current surveys usually face limitations in terms of technical and financial constraints, resulting in the sequencing of only a few hundred to a few thousand individual cells.^106^, ^107^, ^108^ It is unclear how sequenced cells will reflect how they are distributed across the target region. Possible solutions to this challenge are to further increase the throughput of cell capture, such as employing MARS-seq and SPLiT-seq techniques. Alternatively, a combination of high-throughput sequencing of bulk tissue samples and SCS followed by deconvolution analysis could be employed as a potential strategy.^109^

A third challenge is that utilizing a solo method in single-cell DNA and RNA sequencing approaches may lead to erroneous inferences regarding adaptive or acquired evolution. Prospective avenues involve the technological implementation of multimodal analysis for both DNA and RNA at the single-cell level. This substantially aids in realizing whether chemoresistant clones originating from the primary tumor may trigger distant metastases and acquire resistance at these specific organ locations when examining matched metastatic cancers^110^ and can be used for analyzing a more extensive group of patients diagnosed with triple-negative breast cancer.^111^ A fourth challenge is that the comprehensive characterization of heterogeneous tumors requires massive single-cell gene expression profiling initiatives. However, even the third-generation sequencing technology that solves the problems of too short a sequencing length, loss of information, and base mismatches is limited by the activity of DNA polymerase and the high cost. These can only be addressed by further improving the technology's maturity and reducing future costs.^112^ The fifth challenge is that all known eukaryotic scRNA-seq protocols are restricted to detecting poly(A) tailed mRNAs (poly(A) + RNAs). Nevertheless, this method ineluctably excludes information on RNA species such as circRNAs and microRNAs that do not possess poly(A) tails. Recent studies have highlighted the significant impact of non-coding RNAs, including circRNAs and microRNAs, on the development and occurrence of various tumors. As a result, this particular area of research has become a focal point in understanding tumor mechanisms.^113^, ^114^, ^115^, ^116^, ^117^, ^118^ The level of scRNA-seq technology for non-coding RNAs, however, remains consistently suboptimal. Recently, scientists have developed a technique called single-cell universal poly(A) independent RNA sequencing to tackle this problem. This innovative approach allows for the identification of both poly(A)^+^ and poly(A)^−^ RNA within individual cells while effectively minimizing rRNA contamination.^119^ Nevertheless, scRNA-seq containing non-coding RNAs generally remains rare, and its use in tumor research is quite restricted. Generally, further developments and progress in this aspect facilitate the deeper application and underlying clinical significance of scRNA-seq.

## Conclusion

SCS technology can help clinicians guide the selection of targeted therapy by determining the common expression patterns of possible drug targets within cells and tumors. Single-cell expression profiling can uncover the expression of targets, whether they are widespread or limited to a rare subset, and whether possible targets for combination therapy are expressed in a common way or a different subset. The application of SCS technology has had a tremendous impact on cancer research. By sequencing the DNA and RNA of single cells, we can explore the intra-tumoral heterogeneity of primary tumors, track the evolution of drug resistance, and analyze the distinct tumor microenvironment. The implementation of SCS technology can make more groundbreaking biological discoveries at the cellular level, opening up new perspectives for the study of tumor cell subtypes and the overall microenvironment. SCS technology expands the way to explore many mysteries in cancer through the improvement of existing sequencing platforms, the emergence of new technologies for spatial transcriptome sequencing, and its combination with other experimental protocols. The primary focus for advancing SCS technology should concentrate on three aspects, namely, the exploration of single-cell epigenomics, the integration of multiomics, and the advancement of spatial SCS techniques, and advancing SCS technology is expected to bring a new revolution in cancer research for researchers.

## Author contributions

WYGZ, XZ, and FFT wrote the main manuscript text. QJY, JYW, BS, JL, JYZ, XMS, HQZ, and YXX searched the literature, prepared Figure 1, Figure 2, Figure 3, Figure 4, and drew the graphical abstract. KLL and XZW reviewed and revised the manuscript and wrote the guidance. All authors read and approved the publication of the final version of the manuscript.

## Ethics declaration

This article does not contain any studies with patients or animals performed by any authors.

## Data availability

All data are available upon reasonable request from the authors.

## Conflict of interests

The authors declared no conflict of interests in this study.
