# Supplementary material for: Research progress and the prospect of using single-cell sequencing technology to explore the characteristics of the tumor microenvironment
Source: Genes Dis. 2024 Feb 3;12(1):101239. doi: 10.1016/j.gendis.2024.101239 (PMC11566696; doi:10.1016/j.gendis.2024.101239)
Supplement: Multimedia component 1 [file mmc1.doc]

**Summary:**

**Graphical Abstract**

**
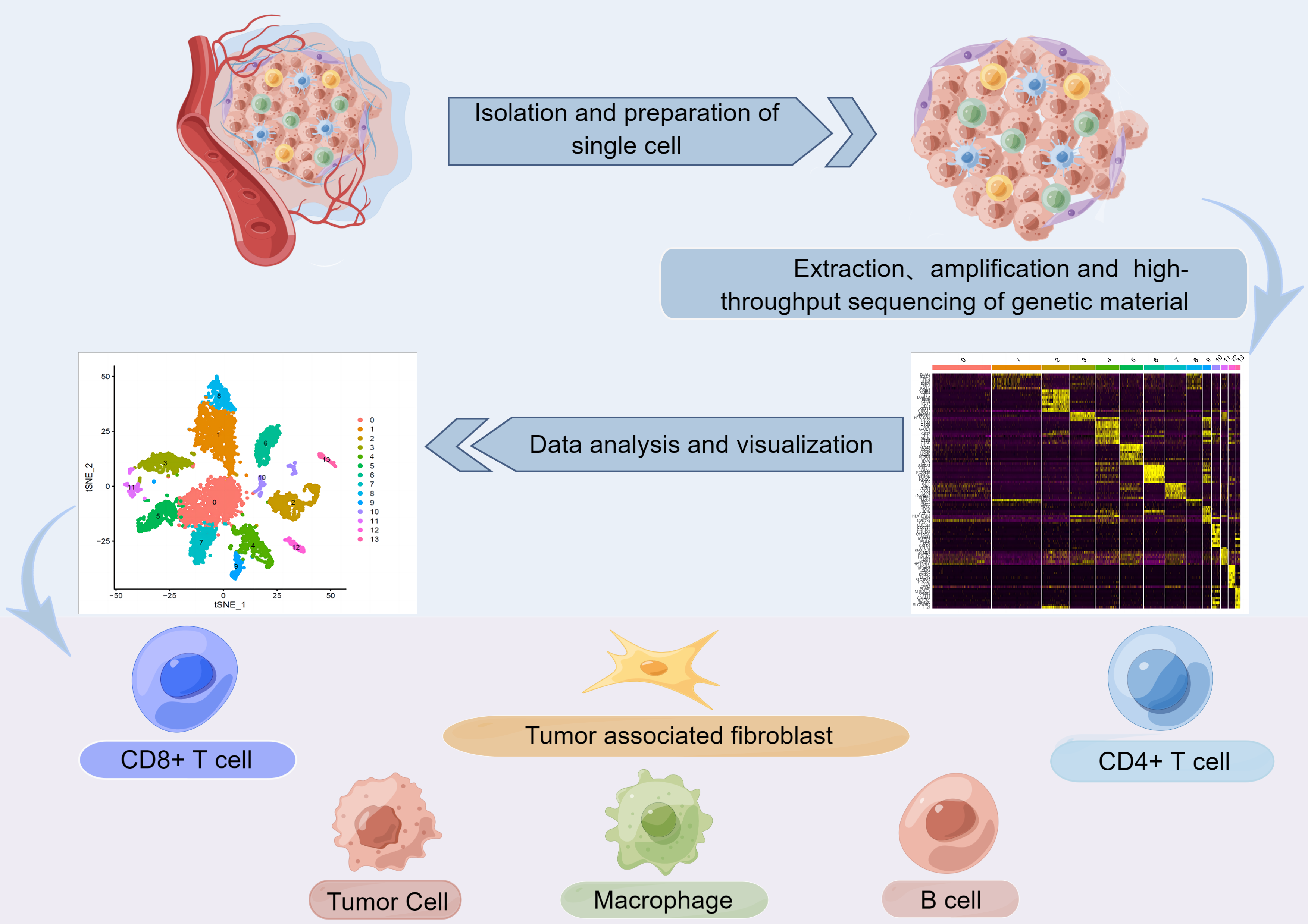
**

**Background:**

The tumor microenvironment (TME) consists of tumor cells and their survival sites, including tumor-associated macrophages, tumor-associated fibroblasts, myeloid suppressor cells, dendritic cells, angiogenic factors, and cytokines (Figure 1). Tumor microenvironment and tumor cells are an interdependent and mutually promoting whole, and their existence provides the necessary material basis for tumor occurrence, development, invasion, metastasis, resistance to drug therapy and immune response. Therefore, in addition to the traditional treatment methods, studying tumor microenvironments is a new way to explore tumor therapy. The scientists found that TME may consist of several lineages of immature, stem-like subpopulations, more differentiated cells, and populations of infiltrating immune cells with individual differences.


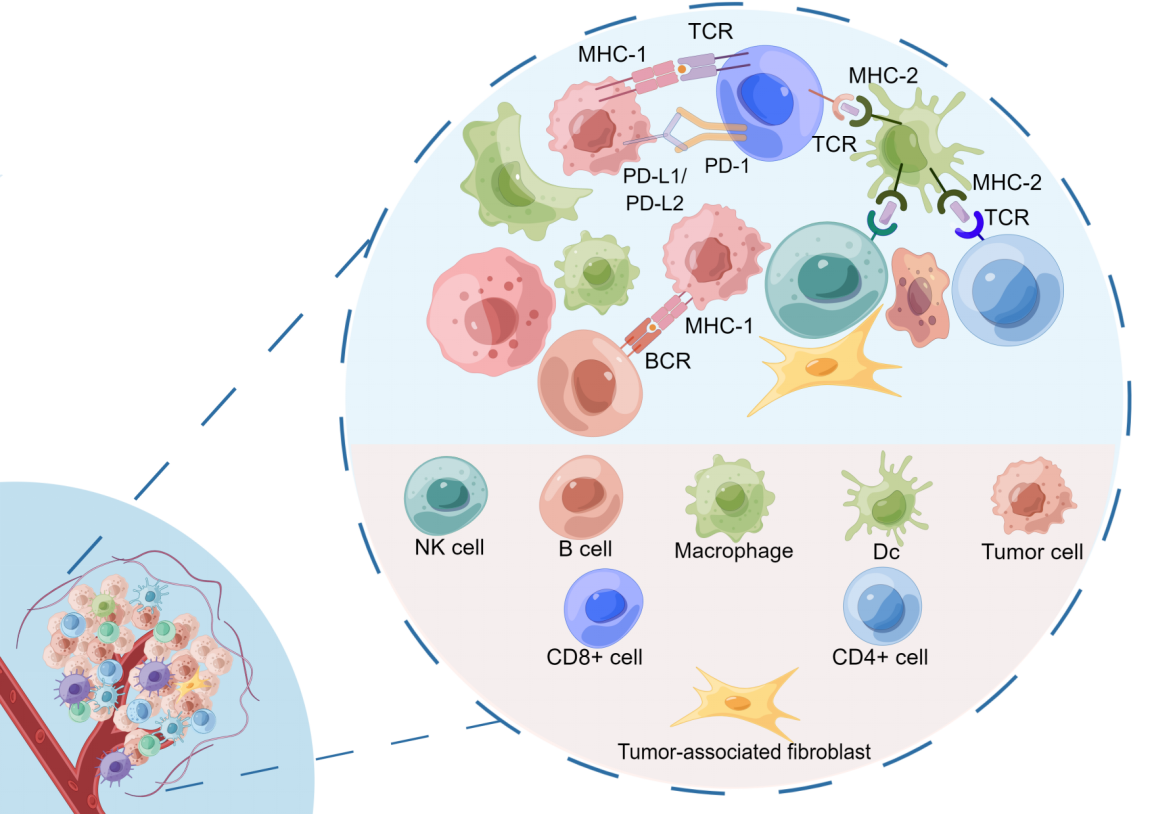


Standard second-generation sequencing can only be performed through blind sequencing, and the heterogeneity of patients' tumors cannot be explored. The research level of single-cell sequencing technology is a single cell, which can isolate and screen single cells and identify and sequence cell populations. Because of its unique cell screening and identification characteristics, it has been widely used in the study of tumor heterogeneity and tumor immune microenvironment. In recent years, single-cell sequencing technology has made remarkable achievements and has been widely used in cancer research, but there are still many challenges and problems to be explored.

**Content:**

In summarizing the progress of single-cell sequencing technology in the tumour microenvironment study, we mainly discuss the most commonly used single-cell genome sequencing and transcriptome sequencing technology.

**Single cell genome sequencing**

The study of the clonal and subclonal formation of primary tumors was an early application of single-cell sequencing in cancer research. Breast cancer, kidney cancer, bladder cancer, colon cancer, hematologic malignancies and glioblastoma have all been treated with DNA single-cell sequencing technology. Single-cell DNA sequencing is primarily used in breast cancer studies to identify somatic nucleotide variants (SNVs), copy number alterations (CNAs), and structural variants (SVs). Currently, the primary method of breast cancer single-cell genome sequencing is to sequence copy number alterations (CNAs) using whole genome sequencing (WGS), which can identify tumor heterogeneity and clonal tissue and progression. In a colon cancer study, Liu M. et al. utilized fluorescence-activated cell sorting techniques followed by low coverage single-cell whole genome sequencing (WGS) at 0.1x depth, allowing them to distinguish individual cancer stem cells from diffuse tumor cells based on somatic cell copy number alterations (SCNAs). Studies of haematological malignancies, including acute lymphoblastic leukaemia and acute myeloid leukaemia, have demonstrated common mutations between cancer cell clones in individual cancer patients.

**Single cell transcriptome sequencing**

In the research progress of single-cell transcriptome sequencing technology, we mainly summarized the application of single-cell transcriptome technology in immune cells, tumor cells and the expression of cell surface markers. The study found that ScRNA-seq is essential in discovering previously unheard-of immune cell types or heterogeneity within the same population of immune cells. One of the more representative examples is that in 2017, Zheng et al. explored the tumor immune microenvironment of liver cancer through single-cell RNA sequencing technology. They found that liver cancer-enriched clonal CD4+CTLA4+ Tregs and depleting CD8+LAYN+ lymphocytes expressed the same TCR. CD8+LAYN+ lymphocytes are derived from CD8+GMZK+ lymphocytes. The HCC microenvironment also includes myeloid suppressor cells with vital immunosuppressive function and tumor-associated macrophage-like cells with an intermediate pro-inflammatory immunosuppressive phenotype and expressing TREM2 (Figure 2). Significant progress was made in its application in 2018, including T-cell immune profilings of lung and colorectal cancer at the individual cell level were analyzed by Professor Zhang's team; researchers at Memorial Sloan-Kettering Cancer Center in the United States have concluded that the tumour microenvironment is formed by less differentiated or activated discrete states, and Dr. Hanjie Li's team at the Ido Amit Laboratory in Israel has contributed to a detailed immune cell map of melanoma.


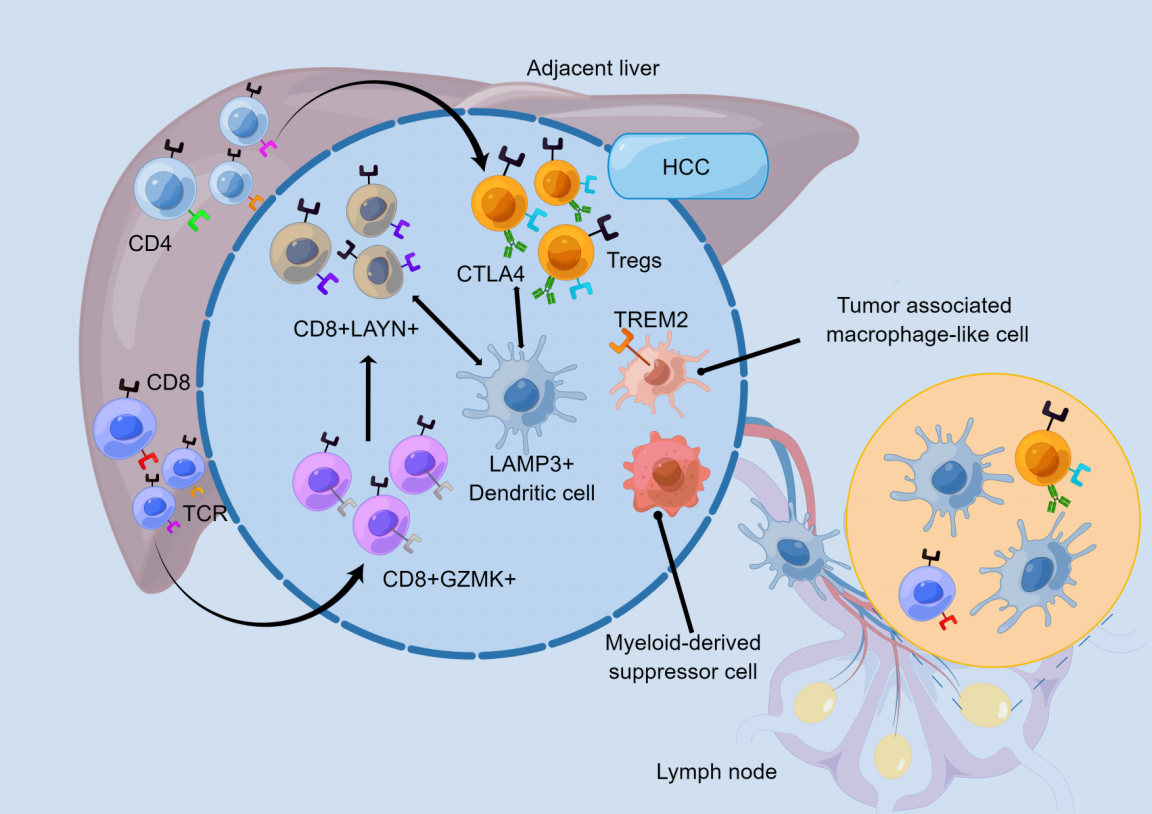


Single-cell transcriptome sequencing of tumor cells was used to study tumor evolution patterns, supporting tumor stem cell models. Applying scRNA-seq to glioma and clonal and subclonal tumor structural features suggested that tumor heterogeneity can be attributed to the cell differentiation process of tumor stem cells. Therefore, it can facilitate the construction of tumor stem cell models. Single-cell transcriptome sequencing can also distinguish between malignant and non-malignant cells in the complex tumour tissue environment. Using scRNA-seq, large CNVS and aneuploids can be quickly identified and used to distinguish cancerous and benign tumor cells.

In 2016, by analyzing the expression of surface markers, researchers found significant heterogeneity in CD127 + innate lymphocytes in the human tonsillar and small intestine. The transcriptome sequencing of single γδ T cells in mouse peripheral and tumor tissues preliminarily demonstrated the diversity of γδ T cells in the tumor microenvironment, the correlation between tumor immune escape and the expression of specific genes in severely damaged immune cells. Using 10X Genomics' single-cell study in lung cancer, the scientists identified the characteristics of each B-cell cluster. Studies have found that attractive biomarkers provided by T-cell cluster marker genes can predict a patient's response to cancer immunotherapy, such as Layilin (LAYN), and these biomarkers may become new targets for further research. Cancer-associated fibroblasts (CAF) and immune cells are required for cancer immune escape and metastasis, and multiple studies have shown that CAF is heterogeneous across multiple cancer types. Therefore, we should further investigate the changing characteristics of CAF gene expression.

Single-cell sequencing technology has an unparalleled application prospect in cancer research. Single-cell technology can use tiny amounts of tissue to explore tumor heterogeneity and, therefore, has high possibilities for clinical cancer diagnosis, prognosis, early detection, risk assessment, progression monitoring, and treatment response prediction. With the advancement of technology, single-cell sequencing technology has also made significant progress in epigenomics, proteomics, metabolomics, and microbiome, and their joint application with single-cell genomics and transcriptomics has developed single-cell multi-omics technology, which can obtain more comprehensive cell information and reveal the interrelationship between multiple omics features of cells—further in-depth study of cell function and regulatory mechanisms. Single-cell multi-omics technology has many application prospects in biological research, medical research and other fields.

In addition, single-cell spatial transcriptome sequencing technology, by coupling single-cell RNA sequencing technology with the spatial information of cells to infer the spatial structure of tumors, has become a new application in cancer research; although it has not been widely used, it has an essential significance in cancer biology and therapy. Fluorescence in situ hybridization (FISH), single-molecule fluorescence in situ hybridization (smFISH), immunohistochemistry, laser capture microanatomy, laser scanning microscopy, or in-situ sequencing can all be used to record spatial information about individual cells or essential "anchor genes" and subsequently measure or calculate the spatial structure of individual cells to reveal the spatial heterogeneity of the tumor microenvironment. The clinical application of this technology provides strong support for the clinical targeted therapy of cancer. For example, phenotypic characteristics and heterogeneity were analyzed at the single-cell level for various human skin cancers, interstitial tumours, and Hodgkin lymphoma. Analytical spatial single-cell sequencing can be combined with a microarray-based spatial transcriptomic approach, followed by multimodal crossover analysis methods.

Single-cell sequencing technology has made remarkable achievements in recent years and has played a massive advantage in exploring the tumor microenvironment. It considers the uniqueness of individual cells and inter-cell heterogeneity, helping us more accurately understand cell type and function and enhancing our understanding of cellular diversity. Scientists developed 10×, BD and other platforms. The innovation of the 10X platform is that individual cells are bound to tiny gel beads, forming GEMs, each containing a cell and a unique barcode (Figure 3). It can sequence large numbers of individual cells simultaneously, providing a more comprehensive picture of single-cell transcriptome information. At the same time, using the RNA-Seq technology, researchers can analyze the tumor microenvironment of various cancers such as melanoma, breast cancer, glioblastoma, colorectal cancer, liver cancer, and lung cancer and map relevant tumor cells profiling with unprecedented resolution.


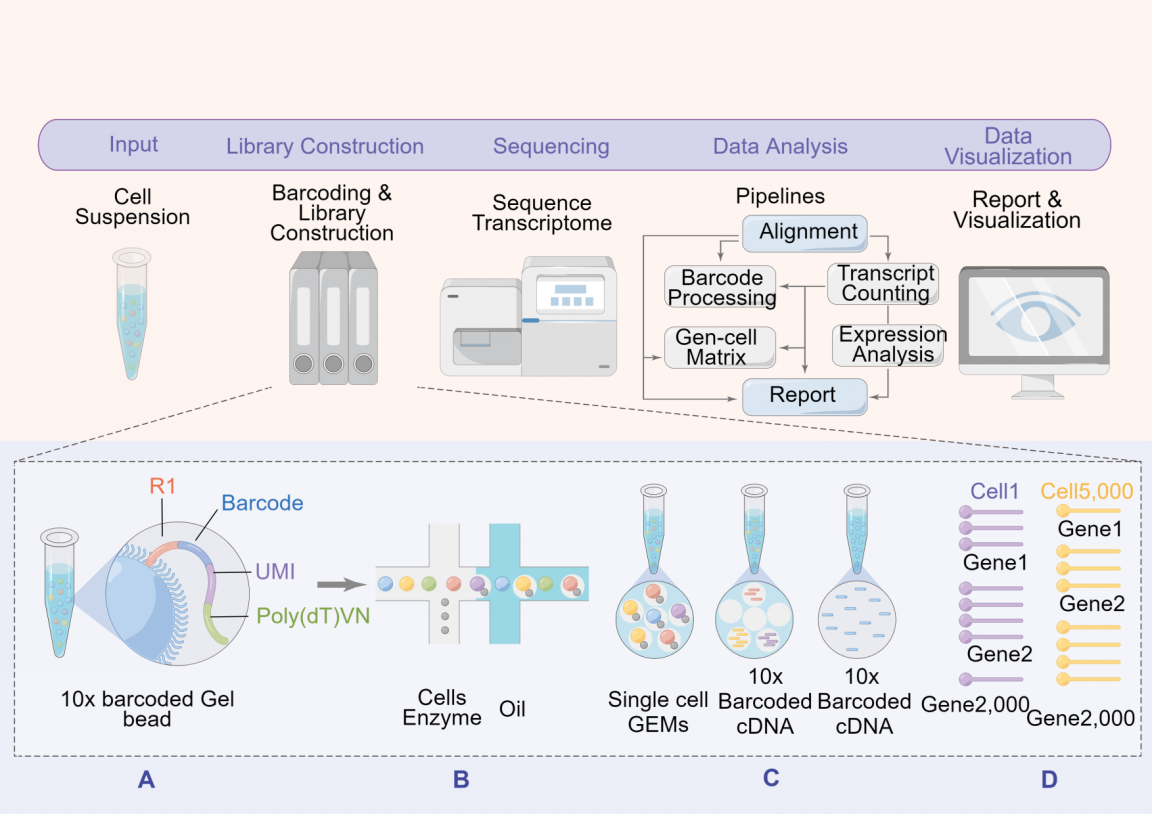


At the same time, single-cell sequencing technology also faces significant challenges (Figure 4). The first challenge is the technical interference introduced during the amplification step. The second challenge is extracting a small number of cells from tumor tissue for sequencing. A third challenge is that applying only one single-cell DNA and RNA sequencing technique can lead to false inferences about adaptive or acquired evolution. The fourth challenge is the comprehensive characterization of heterogeneous tumours requires large-scale single-cell gene expression profiling projects. The fifth challenge is that all known eukaryotic scRNA-seq protocols are limited to detecting poly(A) tailed mRNA (poly(A) + RNAs).


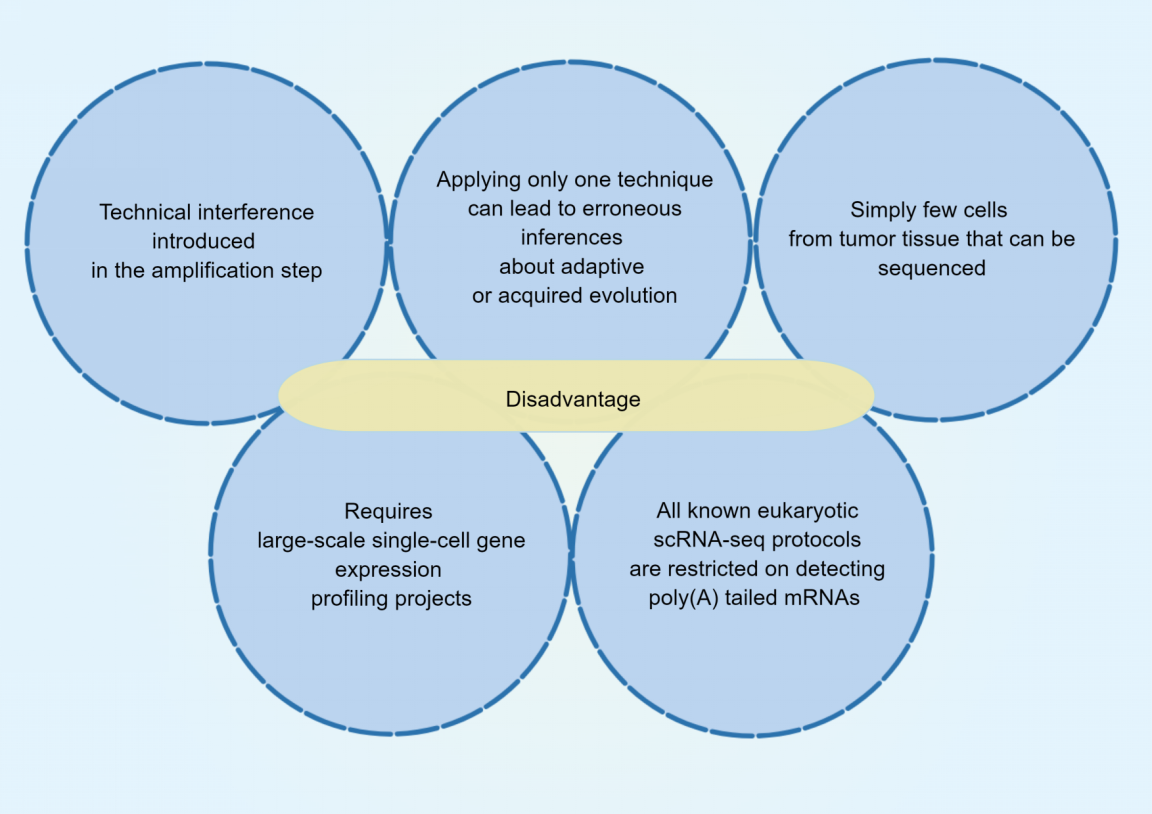


In general, using single-cell sequencing technology to explore the characteristics of the tumor microenvironment is a research area with great potential. Promoting single-cell sequencing technology should focus on three aspects: single-cell epigenomics, multi-omics and spatial single-cell sequencing technology, which is expected to bring a new revolution in cancer research for researchers. By gaining a deeper understanding of how tumor cells interact with their surroundings, we can provide more precise strategies for tumor diagnosis and treatment.

Corresponding author Professor Wang Xiaozhong is currently the director of the Clinical Laboratory Department of the Second Affiliated Hospital of Nanchang University, the deputy director of the Key Laboratory of Molecular Medicine of Jiangxi Province and so on. As a professor in the clinical Laboratory of the Second Affiliated Hospital of Nanchang University, Professor Wang Xiaozhong not only performed well in clinical work, but also committed to scientific research and teaching. He actively participates in scientific research projects and constantly explores new testing methods and techniques to provide better support for clinical practice. The team is mainly composed of undergraduates and postgraduates from the School of Medicine of Nanchang University. We actively participate in scientific research and social practice activities, constantly broaden our horizons and academic abilities. Our team has published many papers, for example: Transcriptomic analysis identifies a pan-cancer association of IL27 expression with cancer prognosis and immune microenvironment; Identification of inflammatory factor-related genes associated with the prognosis and immune cell infiltration in colorectal cancer patients and so on.
